# Supplementary material for: Antigen-Heterologous Vaccination Regimen Triggers Alternate Antibody Targeting in SARS-CoV-2-DNA-Vaccinated Mice
Source: Vaccines (Basel). 2024 Feb 20;12(3):218. doi: 10.3390/vaccines12030218 (PMC10974121; doi:10.3390/vaccines12030218)
Supplement: Supplementary file 1 [file vaccines-12-00218-s001.zip › vaccines-2864627-supplementary.pdf]

Supplementary Material

Antigen heterologous vaccination regimen triggers alternate antibody targeting in SARS-CoV-2 DNA vaccinated mice

Anders Frische, Karen Angeliki Krogfelt, Anders Fomsgaard, Ria Lassaunière*

*** Correspondence:** Corresponding Author: mlas@ssi.dk

1 Supplementary Figures

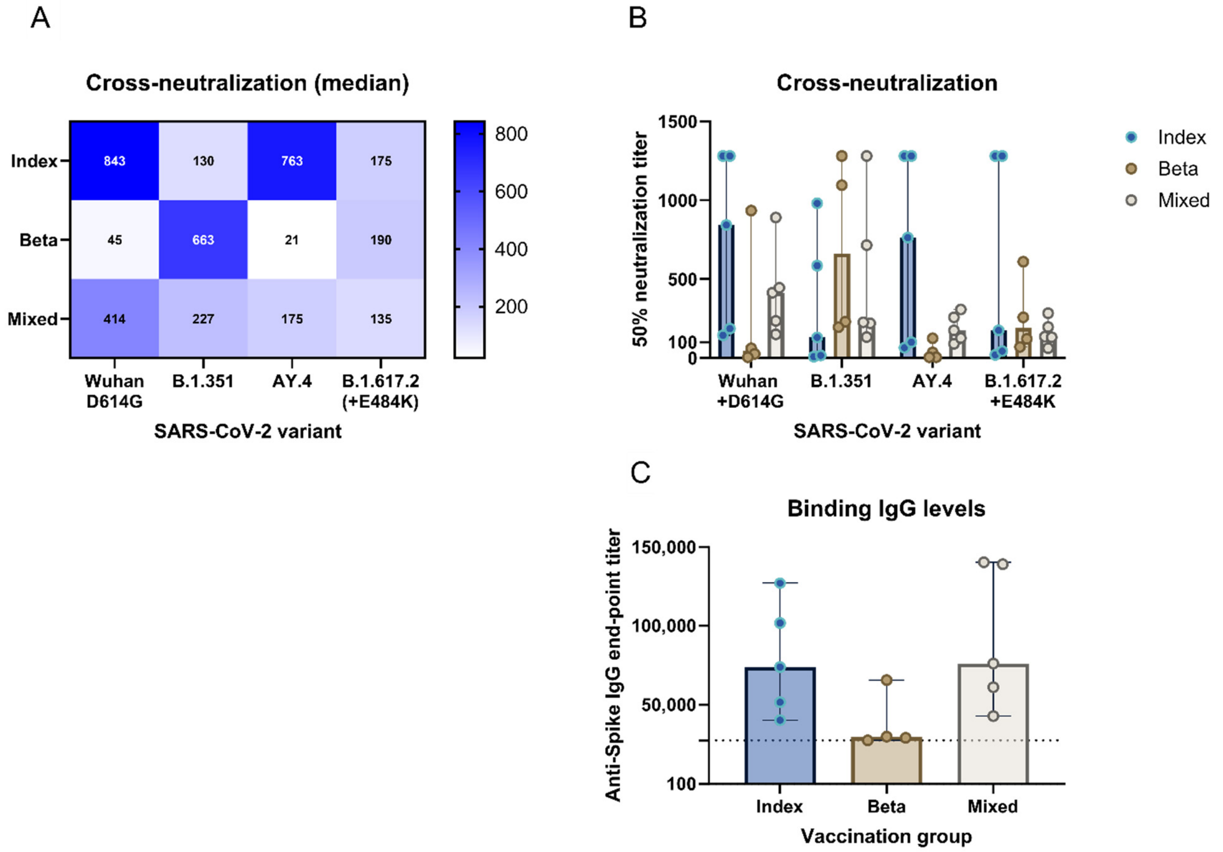

Supplementary Figure S1. Levels of cross-neutralization. Mice received three immunizations with 2-week intervals of a DNA vaccine encoding either the SARS-CoV-2 Index strain spike protein (Index), the SARS-CoV-2 Beta VOC spike protein (Beta) or a combination of the two (Mixed). Two weeks after final vaccination, 50% virus neutralization titers were determined for SARS-CoV-2 variants [1]. (**A**) Heatmap shows median levels of 50% virus neutralization titers. (**B**) Individual levels of cross-neutralization. Bars represent median levels. (**C**) Individual levels of anti D614G Spike IgG (end-point titer) arranged according to vaccination group. Bars represent median levels. Dotted line represents level of normalization.

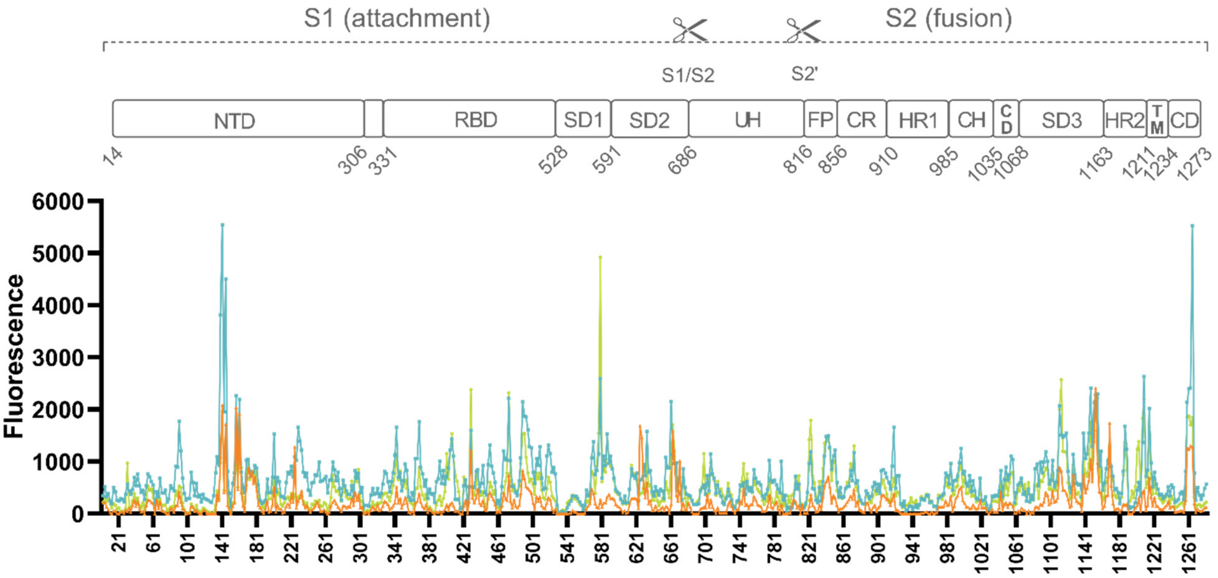

Supplementary Figure S2. Serum binding low affinity antibody profiles. Antibody binding to 10-mer overlapping peptides spanning the ectodomain of the SARS-CoV-2 index strain spike protein with a serum dilution of 1:100 in all three groups. Green: Index vaccinated group; Turquois: Beta vaccinated group; Orange: mix vaccinated group. The x-axis represents the spike protein amino acid as in the last position of the individual peptides. Fluorescence levels on the y-axis indicate the average level of antibody binding to each peptide measured in duplicate and are normalized according to a positive control included on each subarray. S1 subunit (residues 14–685); S2 subunit (residues 686–1273); N-terminal domain (NTD); receptor binding domain (RBD); sub-domain 1, 2, 3 (SD1, SD2, SD3), S1/S2 furin cleavage site; upstream helix (UH); S2' cleavage site; fusion peptide (FP); connecting region (CR); heptad repeat sequence 1 and 2 (HR1, HR2); central helix (CH); connector domain (CD); transmembrane helix (TM); connector domain (CD).

2 References

1. Lassaunière R, Polacek C, Gram GJ, Frische A, Tingstedt JL, Krüger M, Dorner BG, Cook A, Brown R, Orekov T, et al. Preclinical evaluation of a candidate naked plasmid DNA vaccine against SARS-CoV-2. *npj Vaccines* (2021) 6:1–13. doi: 10.1038/s41541-021-00419-z
